# Supplementary material for: Genome-scale community modelling reveals conserved metabolic cross-feedings in epipelagic bacterioplankton communities
Source: Nat Commun. 2024 Mar 28;15:2721. doi: 10.1038/s41467-024-46374-w (PMC10978986; doi:10.1038/s41467-024-46374-w)
Supplement: Supplementary file 1 — Supplementary Information [file 41467_2024_46374_MOESM1_ESM.pdf]

# Genome-scale community modelling reveals conserved metabolic cross-feedings in epipelagic bacterioplankton communities

Nils Giordano[1#], Marinna Gaudin[1#], Camille Trottier[1], Erwan Delage[1], Charlotte Nef[2], Chris Bowler[2,3] and Samuel Chaffron[1,3*]

[1] Nantes Université, École Centrale Nantes, CNRS, LS2N, UMR 6004, F-44000 Nantes, France.
[2] Institut de Biologie de l'École Normale Supérieure (IBENS), École Normale Supérieure, CNRS, INSERM, PSL Université Paris, F-75016 Paris, France.
[3] Research Federation for the Study of Global Ocean Systems Ecology and Evolution, FR2022/Tara Oceans GOSEE, F-75016 Paris, France.

[#] These authors contributed equally to this work.

[*] Contact: samuel.chaffron@cnrs.fr

# Supplementary information

## Supplementary figures:

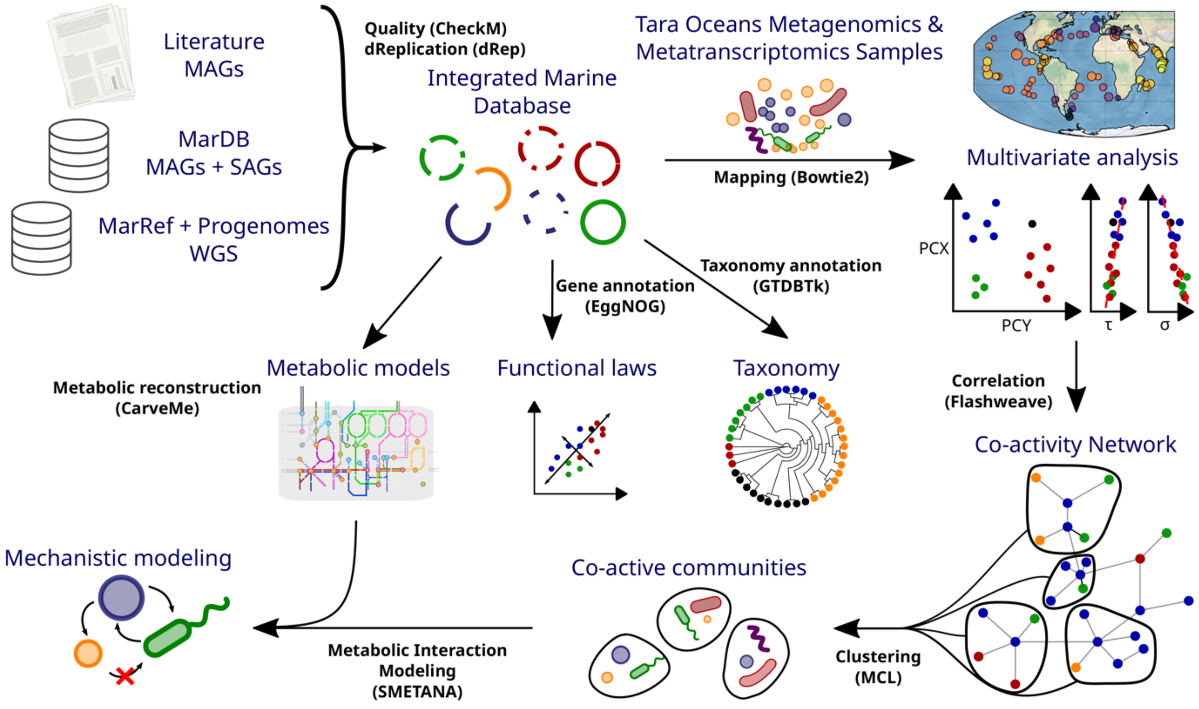


**Supplementary Figure 1: Overview of the computational ecosystems biology workflow.** We developed an integrated ecological and metabolic modelling approach to delineate metabolically cohesive consortia underlying genes-to-community assembly and ecosystem functioning at global scale. Through a multi-omic approach integrating *Tara* Oceans metagenomic and metatranscriptomic datasets, we inferred a global ocean genome-resolved ecological network from whole-genome transcriptomic activities. We used general genomic scaling laws as a

framework to characterise the functional content of co-active environmental genomes, and identified functional gene categories likely driving metabolic dependencies. We then reconstructed genome-scale metabolic models and uncovered putative cross-feeding interactions within co-active consortia through the use of community-level metabolic modelling.

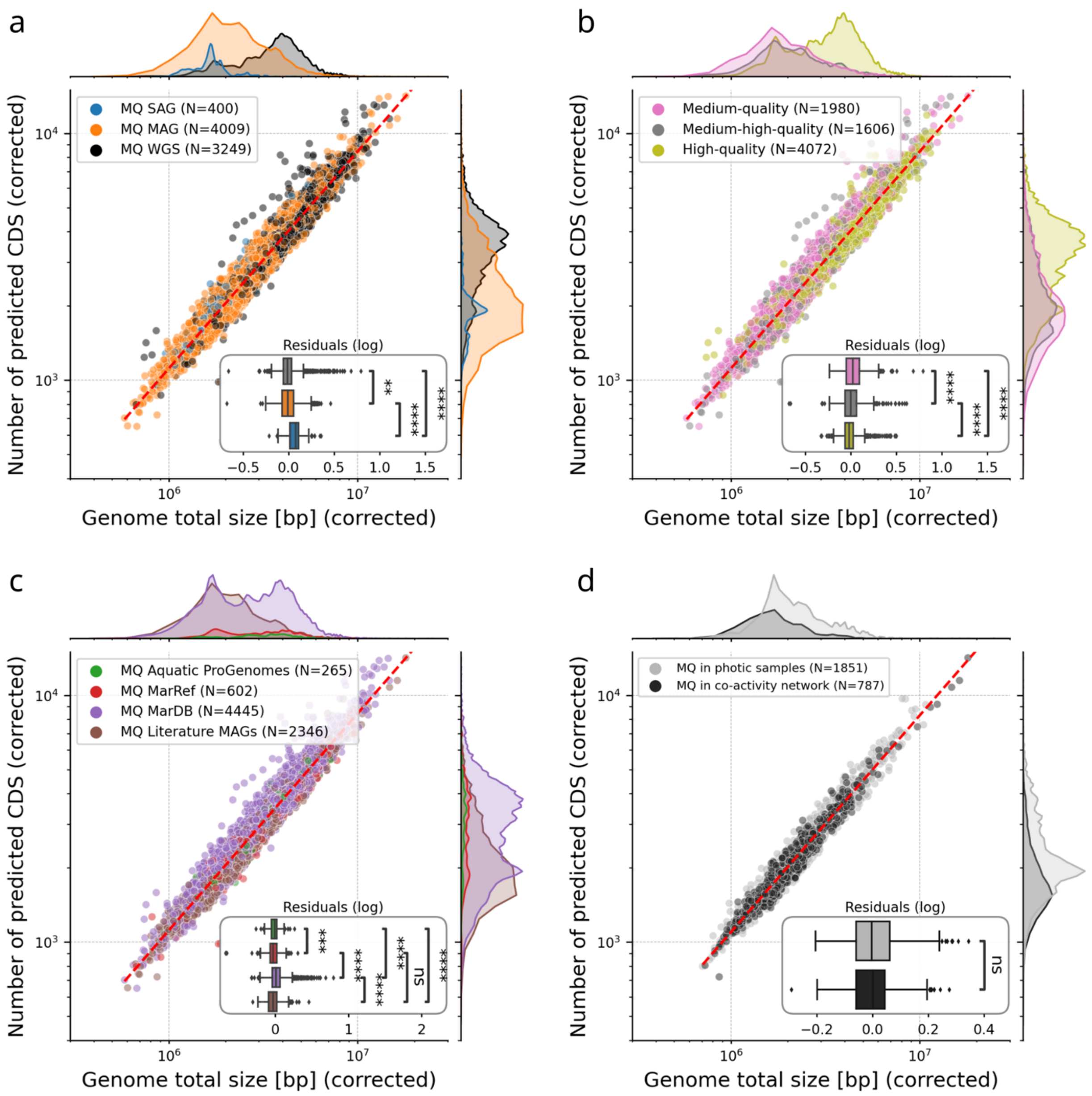


**Supplementary Figure 2: Genomics scaling laws for isolates and uncultivated prokaryotic genomes reconstructed from marine metagenomes.**

Comparison of genome size and number of predicted CDS for Medium Quality (MQ, completeness ≥ 50% and contamination ≤ 25%), and Medium-High Quality (MHQ, completeness ≥ 75% and contamination ≤ 10%) (panel d) dereplicated (95% ANI) genomes. We tested for significant deviations from the common scaling law by **a)** genome type (WGS, SAG, MAG), **b)** genome quality, **c)** source of genome, and **d)** presence in co-activity network (Mann–Whitney U on residuals with Bonferroni correction). Dashed-red lines are the best linear fit on a log-log scale (parameters given in Supplementary Table 3).

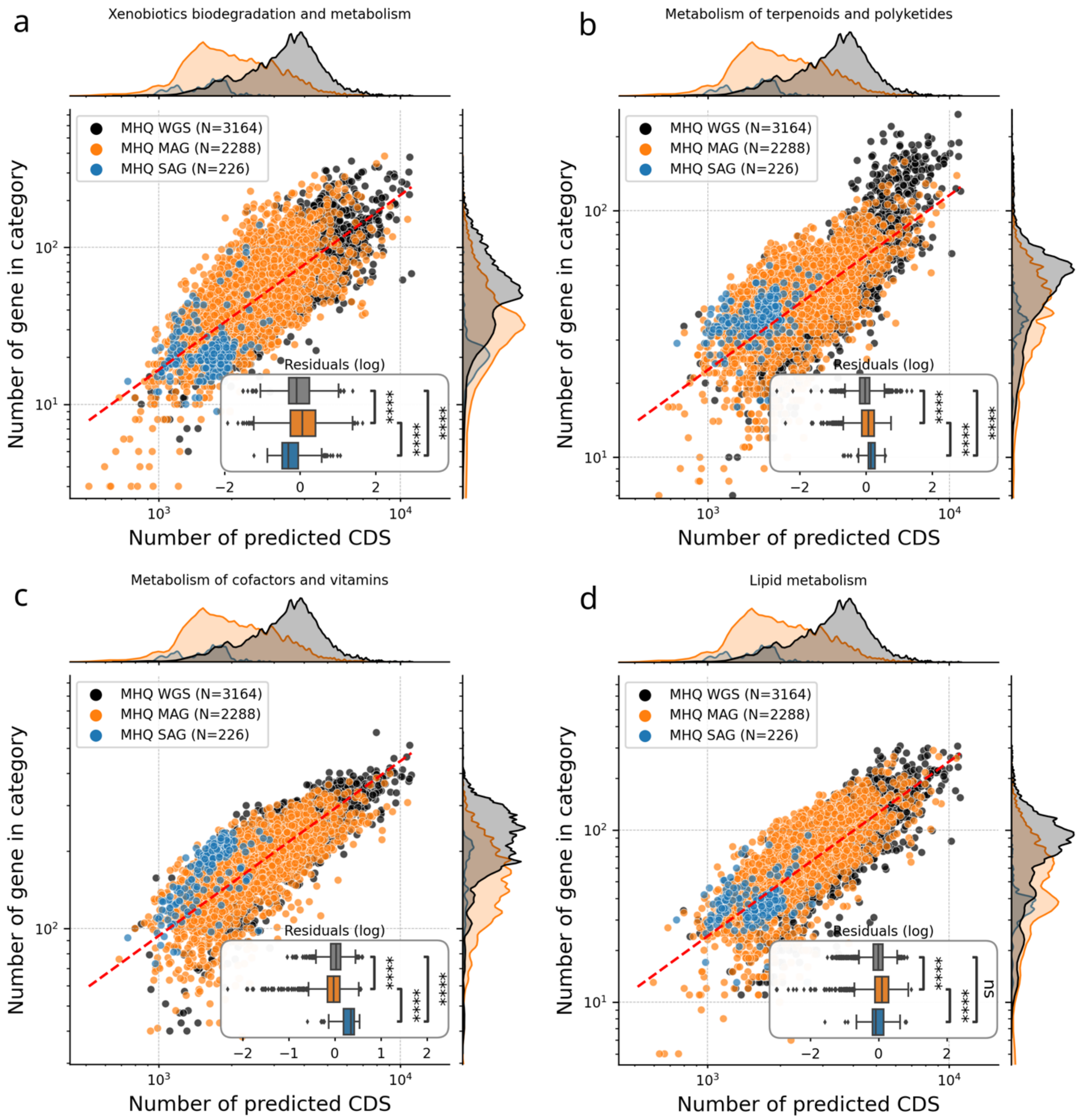


**Supplementary Figure 3: Scaling laws in the functional content of genomes for isolates and uncultivated prokaryotic genomes reconstructed from marine metagenomes.**
Abundance of annotated genes (KEGG database) coding for the metabolism of **a)** xenobiotics biodegradation, **b)** terpenoids and polyketides, **c)** cofactors and vitamins, and **d)** lipids, as a function of the number of CDS for Medium-High Quality (MHQ, completeness ≥ 75% and contamination ≤ 10%) dereplicated (95% ANI) genomes. We tested for significant deviations from the common scaling law by genome type (two-sided Mann–Whitney U on residuals with Bonferroni correction, best fit parameters and *p*-values are described in Supplementary Table 3). Dashed-red lines are the best linear fit on a log-log scale (parameters given in Supplementary Table 3).

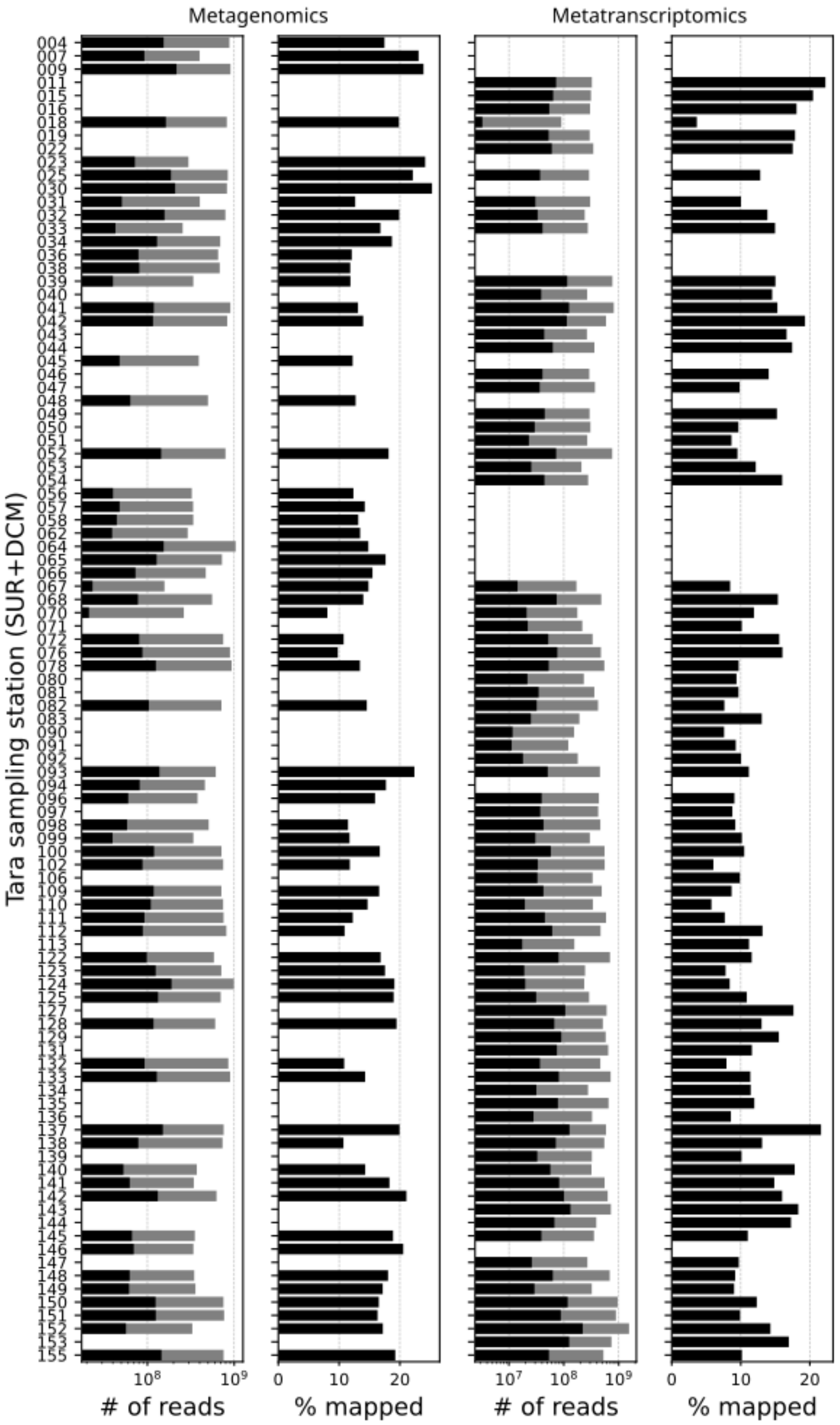


**Supplementary Figure 4: Mapped and Total reads for Metagenomics and Metatranscriptomics across *Tara* Oceans samples.**

Mapping results on the dRep95 catalogue for the metagenomics and metatranscriptomics euphotic samples from *Tara* Oceans expeditions (2009–2013). Black and grey bars are the number of mapped and total reads, respectively. Average mapping rates were 16.0% for metagenomes and 12.3% for metatranscriptomes. We used samples with both metagenomics and metatranscriptomics available to compute genome-wide co-activity.

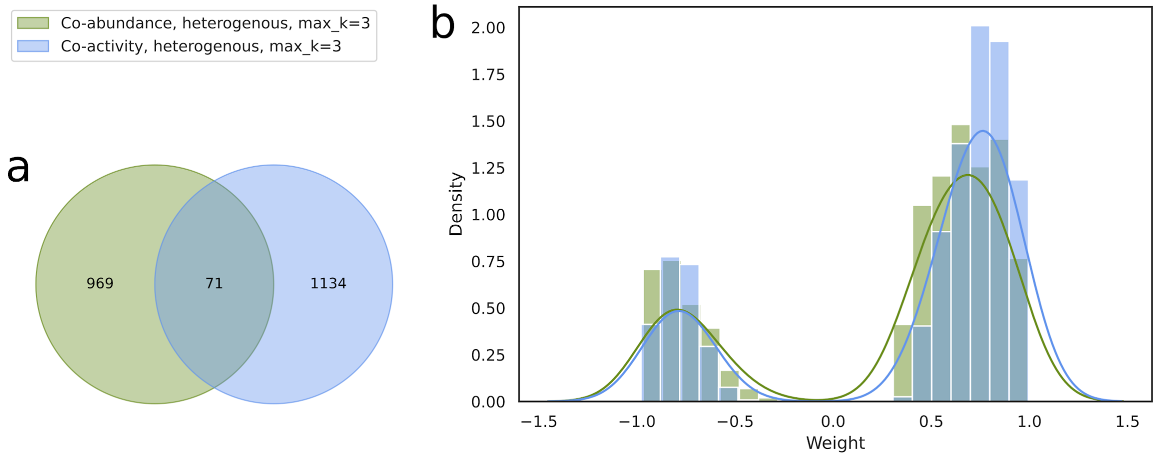


**Supplementary Figure 5: Comparison of genome-resolved co-abundance and co-activity networks.**

**a,** Venn diagram representing the number of shared and unique edges in the global genome-resolved co-abundance and co-activity networks. Only 71 associations were common to both networks, while 1,134 associations are specific to the co-activity network and 969 to the co-abundance network. **b,** Distributions of network weights (inferred by FlashWeave) in both networks. The co-activity network displayed significantly higher weights for positive associations as compared to the co-abundance network (two-sided Mann-Whitney U test, $P < 0.001$).

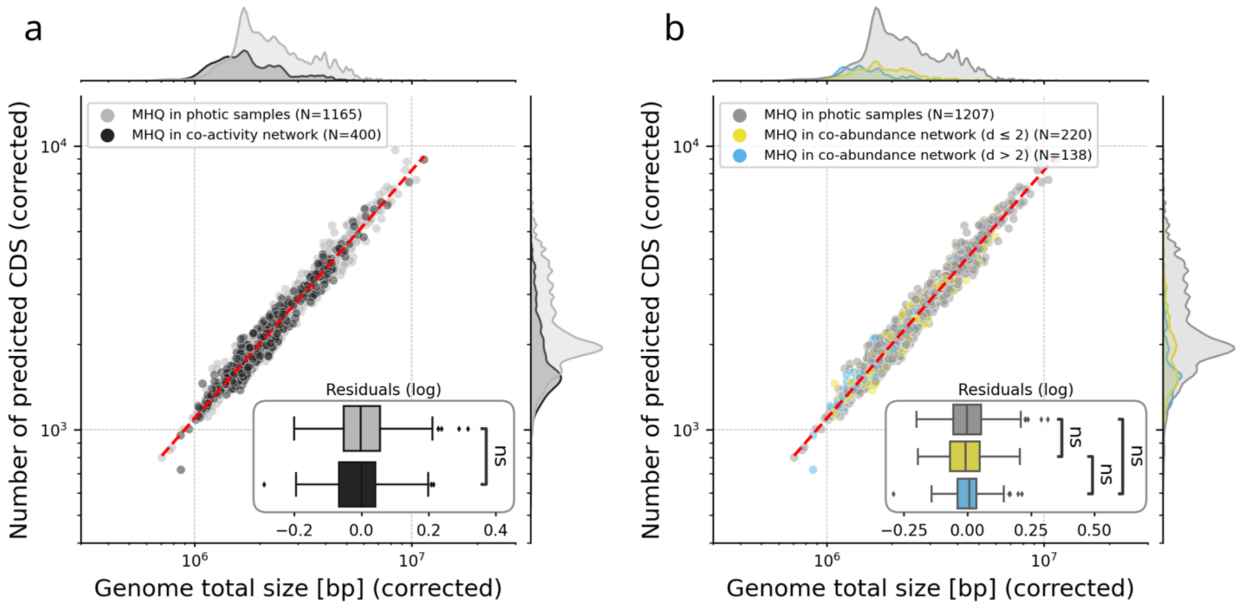


**Supplementary Figure 6: Genomic scaling laws for active and co-active genomes.** Comparison of genome size and number of predicted CDS for Medium-High Quality (MHQ, completeness ≥ 75% and contamination ≤ 10%) dereplicated (95% ANI) genomes. We tested for significant deviations from the represented log-log linear law by **a)** presence in the co-activity network, and **b)** below-median or above-median connectivity degree in the co-activity network (Mann–Whitney U on residuals with Bonferroni correction, best fit parameters and *p*-values are described in Supplementary Table 6). Genomes in photic (SRF and DCM) samples are genomes that were detected active in at least one sample (see Methods). Genomes annotated $d \leq 2$ (respectively, $d > 2$) are genomes with at most 2 connections in the graph (respectively, at least 3 connections). Genomes in the co-activity network are significantly smaller both in size and number of CDS (two-sided Mann–Whitney U test, $p$=1.84 × $10^{-45}$ and 3.22 × $10^{-46}$ respectively). Dashed-red lines are the best linear fit on a log-log scale (parameters given in Supplementary Table 3).

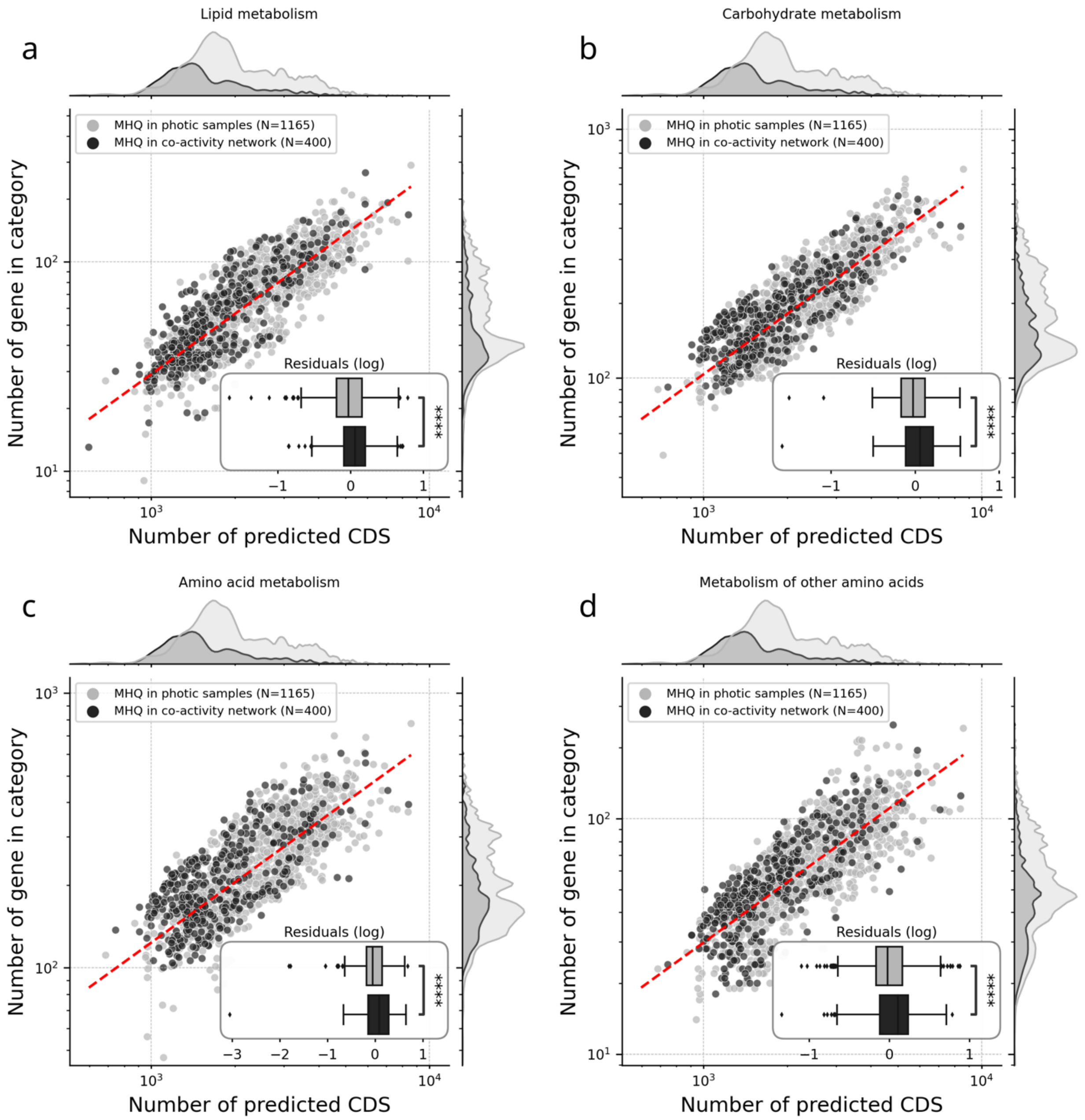


**Supplementary Figure 7: Scaling laws in the functional content of genomes for active and co-active genomes.**

Abundance of annotated genes (KEGG database) coding for the metabolism of **a)** lipids, **b)** carbohydrates, **c)** amino acids, and **d)** other amino acids, as a function of the number of CDS for Medium-High Quality (MHQ, completeness ≥ 75% and contamination ≤ 10%) dereplicated (95% ANI) genomes. Genomes in photic (SRF and DCM) samples are genomes that were detected active in at least one sample (see Methods). We tested for significant deviations from the common scaling law by category of genome (Mann–Whitney U on residuals with Bonferroni correction, best fit parameters and *p*-values are described in Supplementary Table 6).

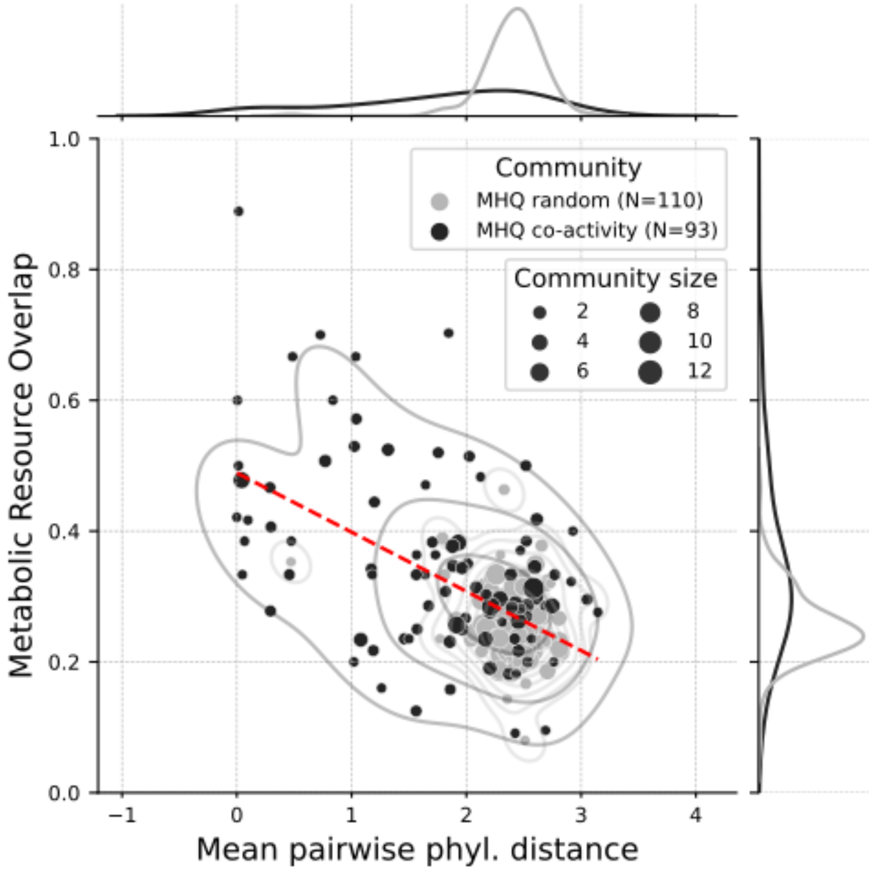


**Supplementary Figure 8: Metabolic resource overlap as a function of phylogenetic distance within communities of co-active genomes.**

Comparison of Metabolic Resource Overlap (SMETANA global score) and Mean Pairwise Phylogenetic Distance for co-active and randomly-assembled genome communities (see methods). The point size refers to the number of genomes in the community. Dashed-red line is the best linear fit and shows a significant negative relationship (slope=–0.091; intercept=0.49; $r^2$=0.31; $p$=4.17 × $10^{-18}$).

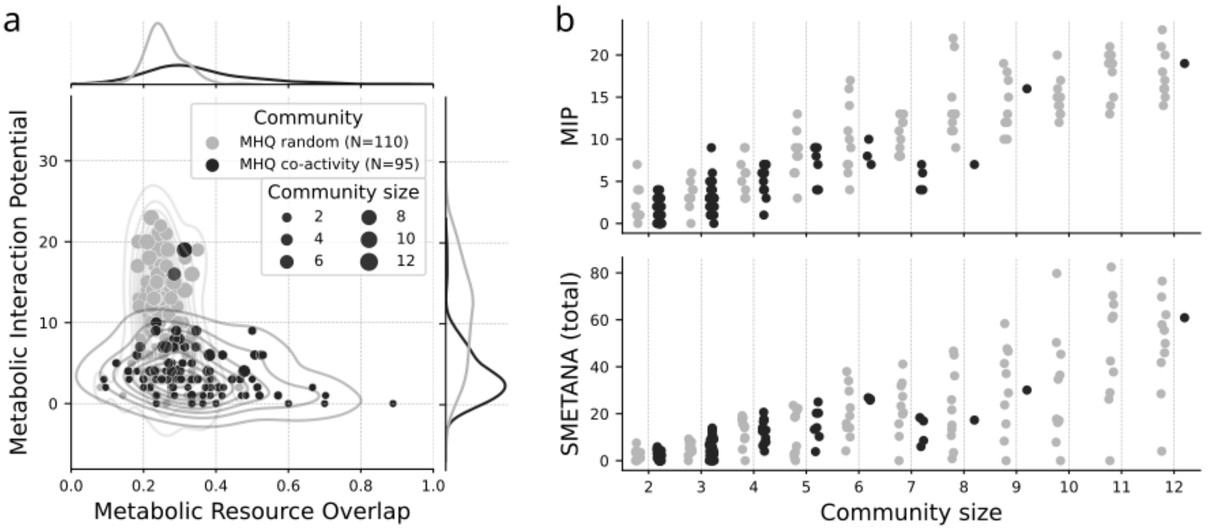


**Supplementary Figure 9: Community-wide metabolic modelling within marine prokaryotic communities.**

**a,** Comparison between Metabolic Interaction Potential (MIP) and Metabolic Resource Overlap (MRO) for co-active and randomly-assembled genome communities. A lower MIP score and a higher MRO score was observed for co-active genome communities as compared with randomly-assembled genome communities. The point size refers to the number of genomes in the community.
**b,** Effect of community size on MIP and SMETANA scores for co-active and random communities. Both scores were significantly driven by community size (MIP $R^2$=0.82, $p$=1.03 × $10^{-77}$; SMETANA $R^2$=0.59, $p$=7.28 × $10^{-41}$).

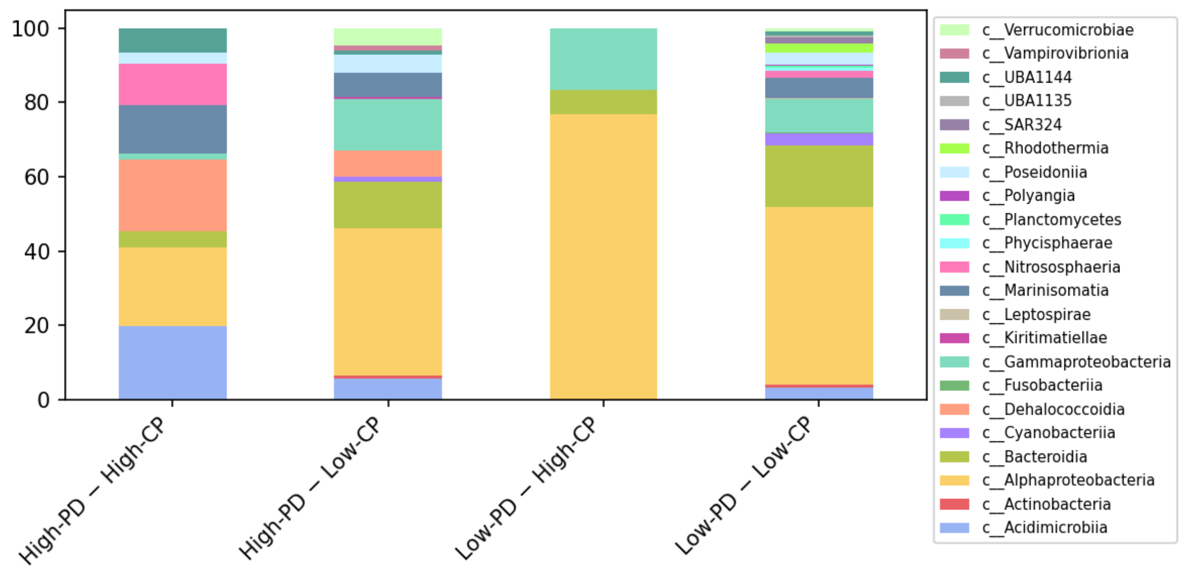


**Supplementary Figure 10: Taxonomic composition of co-active genome community types at the organismal class level.**

The taxonomic composition of the four co-active genome community types is presented as relative proportion at the class-level. The four co-active genome community types displayed distinct taxonomic compositions, with LPD-HCP communities mainly composed of Gamma- and Alphaproteobacteria, while HPD-HCP were more diverse including genomes from classes Nitrososphaeria, Marinisomatia, Dehalococcoidia, Alphaproteobacteria, and Acidimicrobiia. The x-axis corresponds to the proportion taxonomic groups from 0 to 100%.

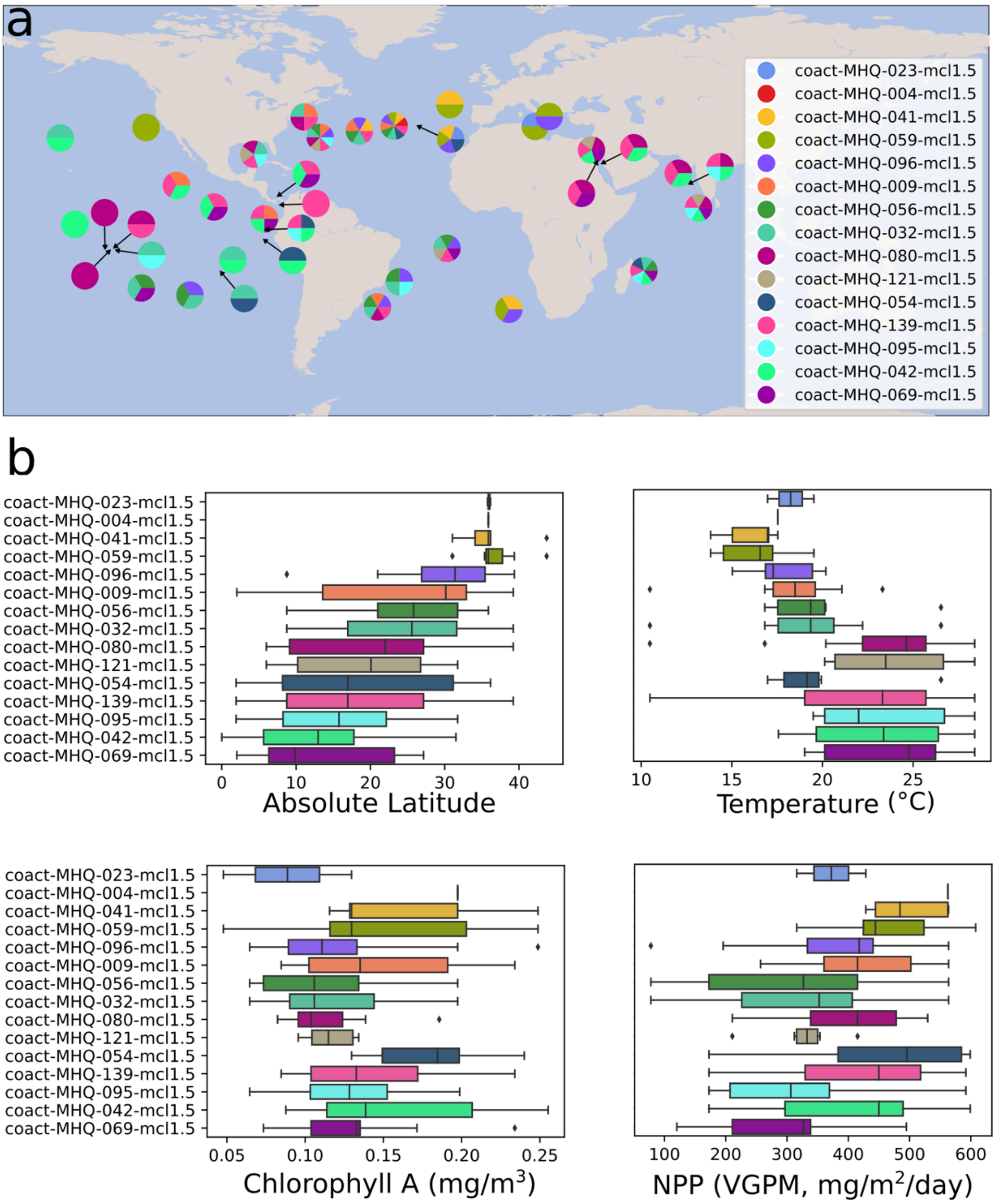


**Supplementary Figure 11: Biogeography of HCP communities and link to productivity.** **a,** The biogeography of HCP communities was analysed based on a presence criteria, with communities being considered present when there were at least 2 genomes for a community size of 2, or a minimum of 3 genomes for a community size greater than 2. Only qualitative information (presence/absence) on the detection of HCP communities is projected on the different stations. **b,** Distributions of environmental variables (absolute latitude, temperature, Chlorophyll A, and Net Primary Production (NPP VGPM)) values per HCP community. In all plots communities are ordered by the median of their observed absolute latitude.

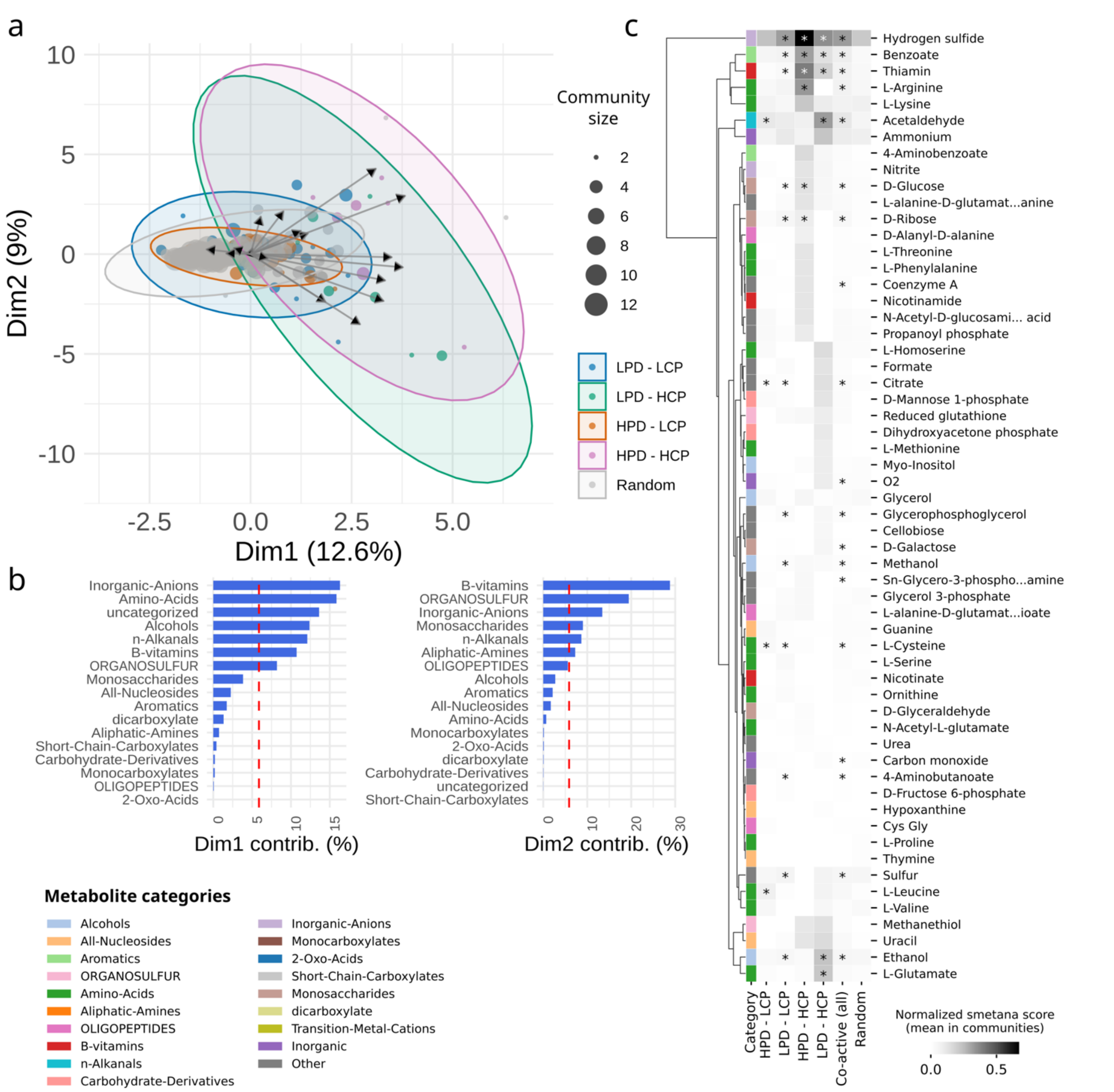


**Supplementary Figure 12: Detailed community metabolic modelling when considering inorganic compounds.**

**a,** NMDS analysis of the co-active genome communities in the space of putative metabolic exchanges (normalized smetana score) for each community type (defined in Fig. 4a). **b,** Contribution of high-level categories of metabolic compounds to the first two dimensions of the NMDS. **c,** Mean normalized smetana score for each high-level category of metabolic compounds in each community type. Similar rows in the heatmap were clustered together by hierarchical clustering (UPGMA algorithm implemented). Truncated metabolite names are (from top to bottom): L-alanine-D-glutamate-meso-2,6-diaminoheptanedioate-D-alanine, N-Acetyl-D-glucosamine(anhydrous)N-Acetylmuramic acid, Sn-Glycero-3-phosphoethanolamine, and L-alanine-D-glutamate-meso-2,6-diaminoheptanedioate.

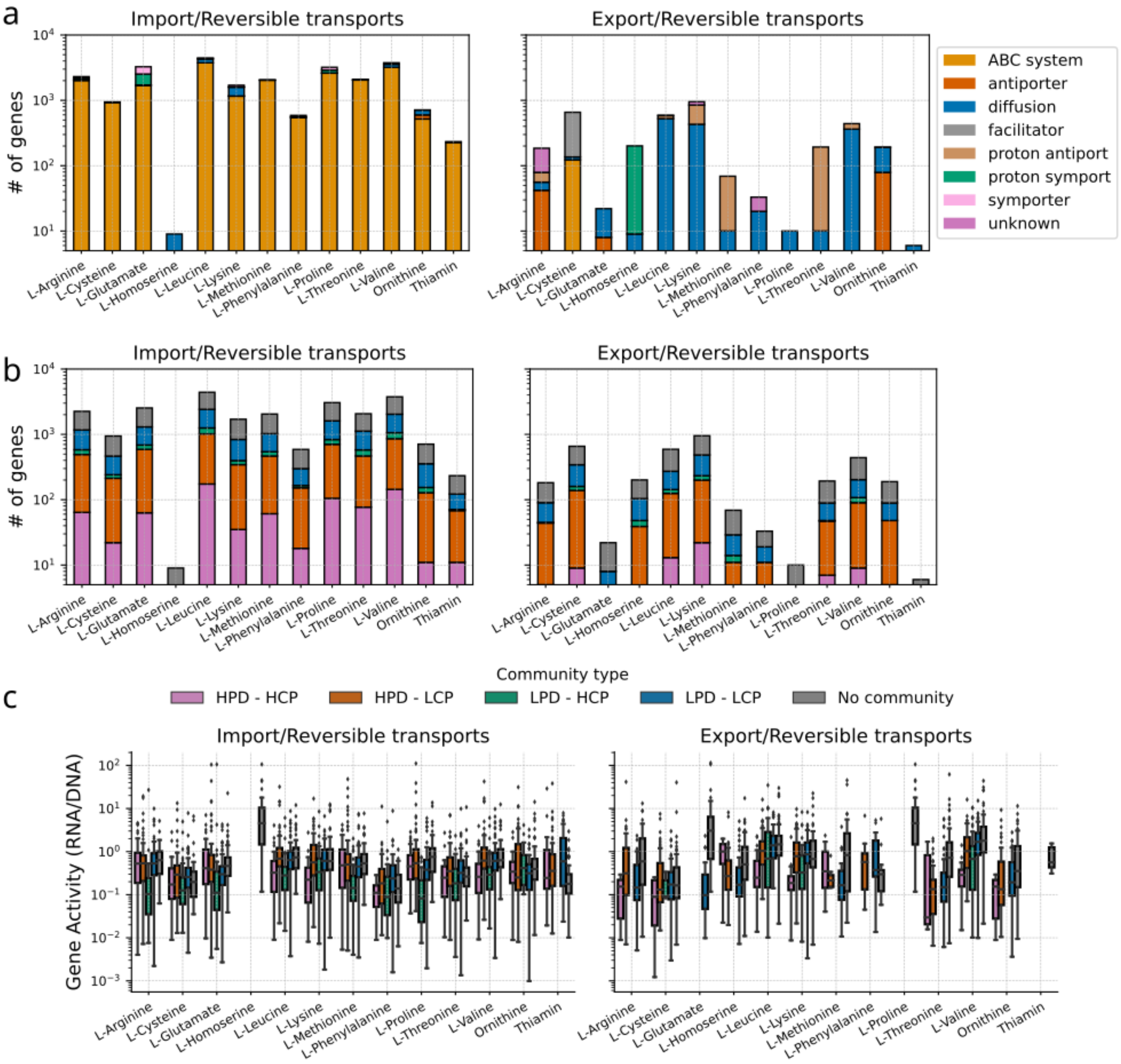


**Supplementary Figure 13. Prevalence and activity of amino acids and B vitamins transporters within genomes of co-active communities.**

**a,** Number of genes for import and export transporter types for amino acids and B vitamins predicted exchanged within genomes of co-active communities. **b,** Number of genes for import and export transporters for amino acids and B vitamins predicted exchanged within genomes by co-active community types. **c,** Gene activities for import and export transporters of amino acids and B vitamins predicted exchanged within genomes by co-active community types. We considered a transporter active if at least one of its components was actively transcribed.

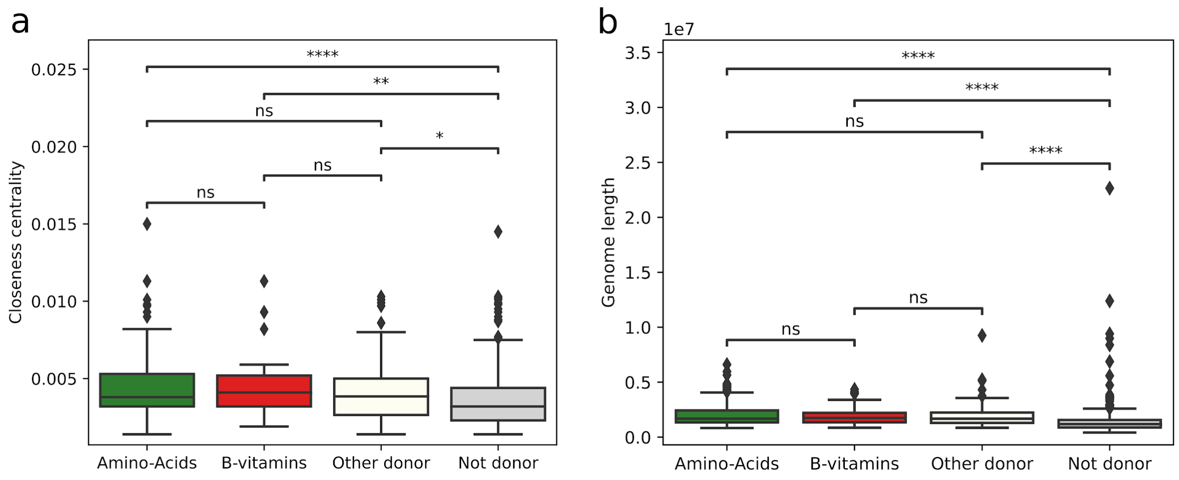


**Supplementary Figure 14. Co-active network closeness centrality and genome size for amino acids donors, B vitamins donors, other compounds donors, and non-donors.**
**a,** Closeness centrality estimates how fast the flow of information would be through a given node to other nodes. All categories of donors had a significantly higher closeness centrality index as compared to non-donors in the co-activity network (two-sided Mann-Whitney U test, Benjamini-Hochberg correction). **b,** Similarly, all categories of donors had significantly higher genome size as compared to non-donors (two-sided Mann-Whitney U test, Benjamini-Hochberg correction). The y-axis for genome length is provided in million bp.

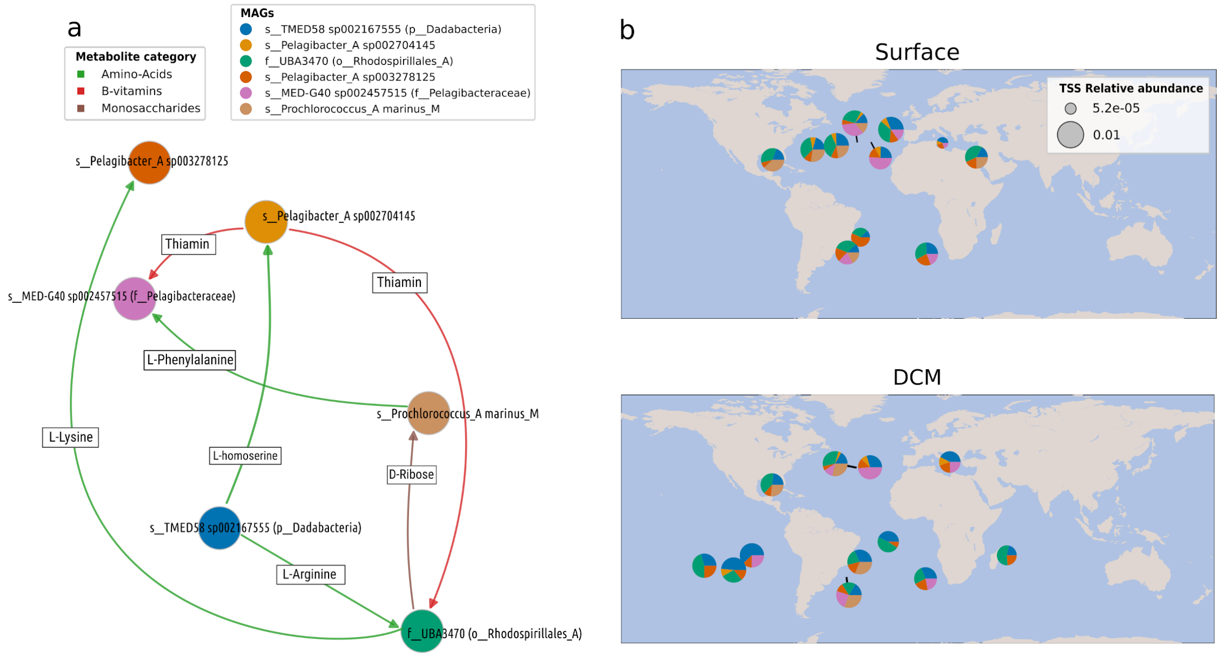


**Supplementary Figure 15. Zooming on a specific co-active genome community including a *Prochlorococcus marinus* genome: Predicted metabolic exchanges and biogeography.**
**a,** Graph representing predicted metabolic exchanges (SMETANA score ≥ 0.5) between genomes of community '*coact-MHQ-014*'. This community included one genome of *Prochlorococcus marinus* (brown), three genomes of Pelagibacteraceae (two Pelagibacter sp. and one MED-G40 sp.; red, gold and pink), one genome of order Rhodospirillales (family UBA3470; green), and one genome of phylum Dadabacteria (TMED58 sp.; blue). Exchanges of several amino acids, B1 vitamin, and D-Ribose were predicted between these genomes. **b,** Biogeography of the respective community '*coact-MHQ-014*' and corresponding genome relative abundances at SRF and DCM *Tara* Oceans stations. The community was considered active if there were at least two genomes and one Pelagibacter detected at each station. The biogeography of this community revealed a globally distributed activity in both SRF and DCM, but restrained to mainly Westerlies (temperate) stations between 30°-60°N/S latitude (mean 33.8°N/27.4°S in SRF, mean 34.3°N/21.7°S in DCM).
