# Supplementary material for: Genome-scale community modelling reveals conserved metabolic cross-feedings in epipelagic bacterioplankton communities
Source: Nat Commun. 2024 Mar 28;15:2721. doi: 10.1038/s41467-024-46374-w (PMC10978986; doi:10.1038/s41467-024-46374-w)
Supplement: Supplementary file 4 — Description of Additional Supplementary Files [file 41467_2024_46374_MOESM4_ESM.pdf]

Genome-scale community modelling reveals conserved metabolic cross-feedings in epipelagic bacterioplankton communities

Nils Giordano[1#], Marinna Gaudin[1,2#], Camille Trottier[1], Erwan Delage[1], Charlotte Nef[2,3], Chris Bowler[2,3] and Samuel Chaffron[1,3*]

[1] Nantes Université, École Centrale Nantes, CNRS, LS2N, UMR 6004, F-44000 Nantes, France.
[2] Institut de Biologie de l'École Normale Supérieure (IBENS), École Normale Supérieure, CNRS, INSERM, PSL Université Paris, F-75016 Paris, France.
[3] Research Federation for the Study of Global Ocean Systems Ecology and Evolution, FR2022/Tara Oceans GOSEE, F-75016 Paris, France.

[#] These authors contributed equally to this work.

[*] Contact: samuel.chaffron@cnrs.fr

Description of Additional Supplementary Files

Supplementary data legends:

File name: Supplementary Data 1

Description: List of all genomes (WGS, MAG, SAG) included in the study.

File name: Supplementary Data 2

Description: Genome summary statistics.

File name: Supplementary Data 3

Description: Scaling law-based comparative statistics for genome types.

File name: Supplementary Data 4

Description: Tara Oceans samples contextual parameters (metadata).

File name: Supplementary Data 5

Description: Principal Coordinates Analyses correlative statistics.

File name: Supplementary Data 6

Description: Scaling law-based comparative statistics for (co-)active genomes.

File name: Supplementary Data 7

Description: List of predicted metabolic exchanges in each co-active community.

File name: Supplementary Data

Description: Statistics of predicted metabolic exchanges in each co-active community type.
